# Fluoride Release from Two Commercially Available Dental Fluoride Gels—In Vitro Study

**DOI:** 10.3390/gels11020135

**Published:** 2025-02-14

**Authors:** Paweł J. Piszko, Aleksandra Piszko, Sylwia Kiryk, Jan Kiryk, Julia Kensy, Mateusz Michalak, Jacek Matys, Maciej Dobrzyński

**Affiliations:** 1 Department of Pediatric Dentistry and Preclinical Dentistry, Wroclaw Medical University, Krakowska 26, 50-425 Wroclaw, Poland; 2 Dental Surgery Department, Wroclaw Medical University, Krakowska 26, 50-425 Wroclaw, Poland; 3 Faculty of Dentistry, Wroclaw Medical University, Krakowska 26, 50-425 Wroclaw, Poland; 4 Medical Center of Innovation, Wroclaw Medical University, Krakowska 26, 50-425 Wroclaw, Poland

**Keywords:** fluoride prophylaxis, commercial dental biomaterials, dental fluoride gels, fluoride release, in vitro

## Abstract

Fluoride has remained the most important ingredient in the prevention of tooth decay for many years. Therefore, fluoride prophylaxis should be highly individualized to provide patients with maximum benefits while minimizing the risk of toxic effects. This study aims to compare the degree of fluoride ion release from two commercially available dental fluoride gels (Fluormex and Fluor Protector Gel) in five different physiological solutions as well as their effect on pH. The concentration of fluoride ions and pH of tap water, distilled water, demineralized water, NaCl, and artificial saliva were evaluated before and after 48 h after dissolving and incubating the same amounts of gels. The concentration of fluoride ions was higher in solutions containing Fluormex than Fluor Pro-tector Gel (*p* < 0.05), with the highest concentration in demineralized water (16,917 ppm). It was accompanied by a decrease in pH below the critical value of 5.5 in all solutions except tap water. Not only the composition of the gel but also the chemical composition of the environment affects the release of fluoride ions. No relationship was found between the change in pH and the concentration of fluoride ions.

## 1. Introduction

Fluoride has played a pivotal role in the advancement of dental public health since its initial discovery as a caries-preventive agent in the mid-20th century. The introduction of water fluoridation programs is widely regarded as one of the most significant public health achievements, leading to a marked reduction in caries [1]. However, the use of fluoride has not been without controversy, with debates surrounding its safety, ethical considerations, and the potential for overexposure and overdoses [2]. It is essential to analyze and understand the science behind fluoride’s mechanisms of action, its optimal concentrations, and the balance between benefits and risks in the aim to establish the safe and effective use of fluoride in both individual and community dental care [3].

**Figure 1:**
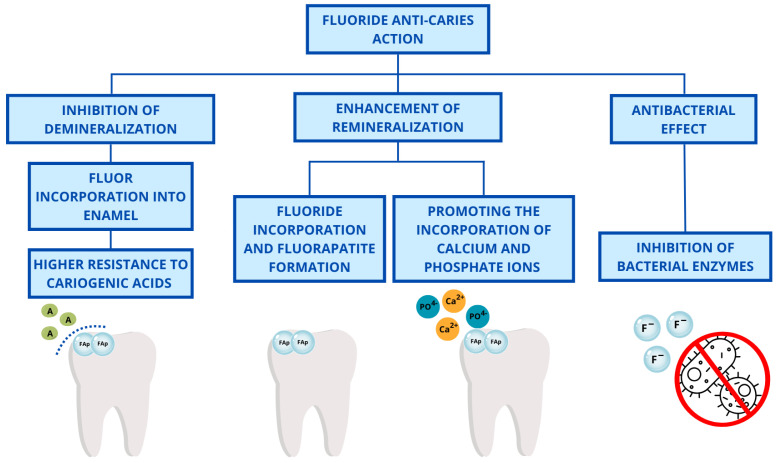
Fluoride delivery methods and its mechanisms of action.

Fluoride compounds are frequently utilized for the prevention, treatment, and inhibition of dental caries, as well as for the prevention of erosion [4,5,6,7]. Fluoride-containing agents can be administered in two principal ways: endogenously, through water fluoridation, ingestion of tablets, consumption of milk, or exogenously, as in the application of pastes, varnishes, foams, or gels [8,9]. Fluoride’s anti-caries action can be defined in terms of two main phenomena: the inhibition of demineralization and the enhancement of enamel remineralization [4,9]. The incorporation of fluoride into enamel apatite in the form of fluorapatite offers significantly more effective protection for tooth tissue from acids in comparison to carboxylated apatite or hydroxyapatite [4,8,10]. The effect of fluoride on remineralization is manifested in the induction of fluorapatite formation and the enhancement of calcium ion attraction [4]. Additionally, fluoride exerts an antibacterial effect by entering bacterial cells as hydrogen fluoride (HF) at a low pH, dissociating within the cytoplasm, leading to intracellular acidification, and releasing fluoride ions. These ions inhibit enolase, a key enzyme in bacterial glycolysis, disrupting metabolism, reducing acid production, and ultimately weakening bacterial survival in the oral environment [1]. The multifaceted action of fluoride is illustrated in the schematic below in Figure 1.

In the context of fluoride compounds, it is imperative to consider both their therapeutic potential and associated risks, particularly fluorosis and fluoride toxicity. Excessive exposure to fluoride during critical stages of dental development can lead to dental fluorosis, characterized by the hypomineralization of enamel, visible discoloration, and, in severe cases, structural damage to the teeth [4,8,11]. This condition arises when children are exposed to fluoride levels exceeding the optimal threshold, whether through fluoridated water, toothpaste ingestion, or dietary sources. Although mild fluorosis is often regarded as an aesthetic concern, severe cases can significantly affect oral health and quality of life [11]. The occurrence of fluorosis may affect both primary and permanent teeth—in the case of primary teeth, the critical moment of excessive fluoride supply is 6–9 months of life, and in the case of permanent teeth—2–8 years of age [12,13]. Consequently, an efficacious and secure therapeutic regimen must carefully consider the patient’s fluoride exposure, caries risk, and age to ensure fluoride compounds are administered safely and effectively [11]. Tailoring fluoride use to individual risk factors—such as age, dietary habits, and overall fluoride exposure—not only mitigates the risk of fluorosis but also maximizes fluoride’s caries-preventive benefits, striking an essential balance between efficacy and safety [14].

Fluoride gels are regarded as preparations for topical use [4]. Their pH ranges from about 3.2 to 7.7 [15]. They can be professionally applied by a dentist, most commonly as 2% acidified phosphate fluoride (APF) with a fluoride concentration of 12,300 ppm or 2% neutral sodium fluoride with a fluoride concentration of 9050 ppm [16]. Fluoride gels are recommended for the prevention and remineralization of carious lesions in the absence of cavities, the reduction of erosion risk in patients at high and moderate risk of caries, the management of dentin hypersensitivity in elderly patients at risk of crown and root caries, and the treatment of fixed orthodontic appliances [5,15,17,18]. The deposition of a calcium fluoride layer on the tooth surface is a consequence of high concentrations of fluoride ions, which are also released into the saliva when the pH drops [19,20]. Low concentrations of fluoride ions have been demonstrated to impede the demineralization process while simultaneously promoting remineralization. In this instance, the reservoir in question is saliva. One of the parameters that indicates the strength of the anti-caries action of a given fluoride-containing agent is the concentration of calcium fluoride formed on the enamel surface [20]. The calcium fluoride formed serves as a reservoir for fluoride ions and calcium, releasing them when the pH of the saliva drops. It has been demonstrated that an increase in the concentration of fluoride gel results in a corresponding increase in the concentration of calcium fluoride, thereby enhancing the efficacy of the treatment [20,21]. The environment in which the fluoride compound is present plays an important role in determining the anti-caries properties of a gel [21]. The utilization of acidified gels (APF) has been demonstrated to result in the formation of a greater quantity of calcium fluoride [22]. The acidic pH facilitates a gradual dissolution of the outer enamel structure, thereby enhancing the absorption of fluoride ions [21,22].

It is also worth noticing that the efficacy of the fluoride-containing compound is contingent upon its stability. Amino fluorides, due to their chemical structure, are less reactive than fluoride ions released from inorganic salts. This is reflected in their effectiveness in preventing and reversing the effects of caries. This does not negate the fact that the fluoride present in both compounds has been demonstrated to possess satisfactory anti-caries potential [21].

Studies on the release of fluoride ions from gels have shown that the type of matrix containing fluoride and the mineral composition of the solvent are important factors influencing the number of released ions [23]. The results of two studies comparing the fluoride release of different types of agents used in caries prevention and treatment, such as foam, APF gel, varnish, and SDF solution, show that there is no statistically significant difference in the number of fluoride ions released in the same solvents at different time intervals [24,25]. A study comparing fluoride release after 24 h, 7 days, and 14 days from APF gel, varnish, SDF solution, and artificial saliva concluded that the gel released the lowest number of ions compared to the SDF solution and varnish [26]. Gels that were not followed by an oral rinse with water, which significantly reduced the fluoride concentration in the subjects’ saliva, had a significantly higher anticaries efficacy. This is an important consideration for people at high risk of caries who need adequate prophylaxis [27]. When utilizing fluoride gels, their effect on salivary pH should be considered. A study by Shakeel et al. [28] showed that 14 days after the application of APF gel, the salivary pH dropped to 4.0. This is below the critical pH of 5.5, below which enamel apatite demineralization occurs [28]. Table 1, with a summary of examples from the literature regarding fluoride gel action, is presented below.

Additionally, scientific research has demonstrated the advantageous effects of integrating excipients into the gel structure to facilitate enamel remineralization. One such excipient is sodium tri-methaphosphate, which resulted in the incorporation of a greater quantity of F-ions into the enamel structure in comparison to a gel lacking sodium trimethaphosphate [20].

The novelty aspect of the presented study regards the evaluation of the fluoride content of two gels with fluoride in different forms (Fluormex—amine fluoride and sodium fluoride; Fluor Protector—potassium fluoride), which has a significant effect on the release of fluoride ions. In addition, the release of fluoride from the two aforementioned commercial fluoride gels was not previously reported in five different physiological solutions (tap water, distilled water, demineralized water, NaCl, and artificial saliva) that differed in chemical composition, which was also an important factor influencing the results of the study.

In light of the above, it is valuable to have knowledge of the fluoride release profile of the utilized commercial fluoride gels in order to anticipate their clinical effect. This study aims to determine the number of fluoride ions released by two commercial fluoride gels in five different physiological solutions and evaluate their effect on the change in pH.

## 2. Results and Discussion

### 2.1. Fluoride Release—Intra-Group Comparison

The concentration of F^−^ anions was evaluated in five different reference solutions: tap water, distilled water, demineralized water, NaCl, and artificial saliva. The median concentration of fluoride ions was 0.20 [0.20–0.23] ppm for tap water and 0.10 [0.10–0.11] ppm for distilled water, while for demineralized water, NaCl, and artificial saliva, the concentration was below the detection limit of the instrument (10^−6^ M) and was therefore considered 0 ppm. Significant differences in the reference group (G1) were found between tap water and demineralized water, NaCl, and artificial saliva (*p* = 0.000042) as well as between distilled water and demineralized water, NaCl, and artificial saliva (*p* = 0.0216).

After 48 h of incubation with the Fluormex gel, the concentration of F^−^ (in ppm) was 7035 [6899–7100] in tap water, 13,666 [12,570–13,900] in distilled water, 16,917 [16,582–17,090] in demineralized water, 9893 [9541–9893] in NaCl, and 13,896 [13,896–13,908] in artificial saliva. A significantly lower concentration of F^−^ was found in the Fluormex tap water solution (G2) compared to distilled water (*p* = 0.006) and demineralized water (*p* = 0.0000). Additionally, the Fluormex solution with demineralized water contained a higher concentration of F^−^ than the solution with NaCl (*p* = 0.0000).

During incubation with Fluor Protector Gel, the concentration of F^−^ (in ppm) was 8824.5 [8764–9177] in tap water, 9188 [9177–9877] in distilled water, 8999 [8564–9141] in demineralized water, 5755 [5529–5790] in NaCl, and 5790 [5790–6008] in artificial saliva. Fluor Protector solutions with tap water, distilled water, or demineralized water were characterized by significantly higher concentrations of F^−^ compared to the solutions with NaCl or artificial saliva (*p* < 0.05). The results of the intra-group comparison are summarized in Table 2.

### 2.2. Fluoride Release—Inter-Group Comparison

**Figure 2:**
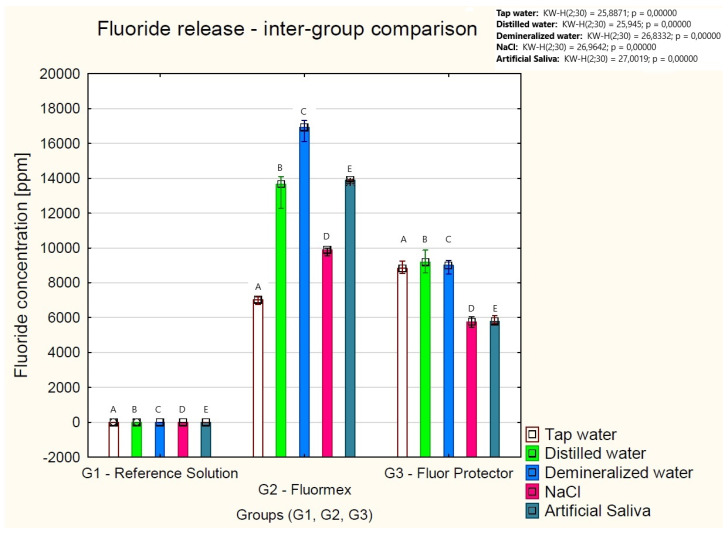
Concentrations of fluoride in reference incubation solutions as well as those of Fluormex and Fluor Protector Gel commercial fluoride gels after 48 h of incubation. The same capital letters (A–E) indicate significant differences in fluoride concentrations among the tested solutions across groups (G1 vs. G2 vs. G3).

The fluoride release in all of the tested solutions (tap water, distilled water, demineralized water, NaCl, and artificial saliva) significantly increased after the addition of the Fluormex and Fluor Protector gels compared to the reference solutions (*p* < 0.001). The addition of the Fluormex gel significantly increased fluoride ion release in distilled water, demineralized water, NaCl, and artificial saliva compared to the Fluor Protector solutions (*p* < 0.05). However, the Fluor Protector solutions with tap water resulted in significantly greater fluoride ion release compared to Fluormex in tap water (*p* < 0.05). The results of the fluoride release are presented graphically in Figure 2.

### 2.3. pH Assessment

The pH levels of the incubation solutions were measured both before incubation and after 48 h of the experiment. Initially (reference solution), the highest pH was recorded in tap water (7.61 ± 0.03), followed by distilled water (7.15 ± 0.07), demineralized water (6.73 ± 0.01), NaCl (6.49 ± 0.04), and artificial saliva (5.04 ± 0.02). After 48 h of incubation with the Fluormex gel, a significant drop in pH was observed in all solutions. The most acidic conditions were found in artificial saliva (4.07 ± 0.04), followed by distilled water (4.69 ± 0.01), demineralized water (4.60 ± 0.02), NaCl (4.99 ± 0.03), and tap water (6.62 ± 0.01). In contrast, incubation with Fluor Protector Gel resulted in either slight increases or smaller decreases in pH levels compared to the initial measurements. Tap water showed the highest pH after incubation (7.84 ± 0.01), followed by demineralized water (6.65 ± 0.04), NaCl (6.62 ± 0.01), distilled water (6.31 ± 0.06), and artificial saliva (5.54 ± 0.01).

A between-group and solution statistical analysis showed significant differences in pH levels, except for the comparison between the G1 and G3 groups for demineralized water and NaCl. Additionally, no significant differences were observed in the pH levels of Fluor Protector Gel (G3 group) solutions when comparing demineralized water, NaCl, and artificial saliva (*p* > 0.05). The results for the pH levels are presented in Table 3.

Fluoride release varies across groups depending on pH (Spearman’s rank correlations test). In the G1 (reference) group, fluoride release was negligible regardless of pH, with pH values ranging from 5.04 ± 0.02 (artificial saliva) to 7.61 ± 0.03 (tap water).

**Figure 3:**
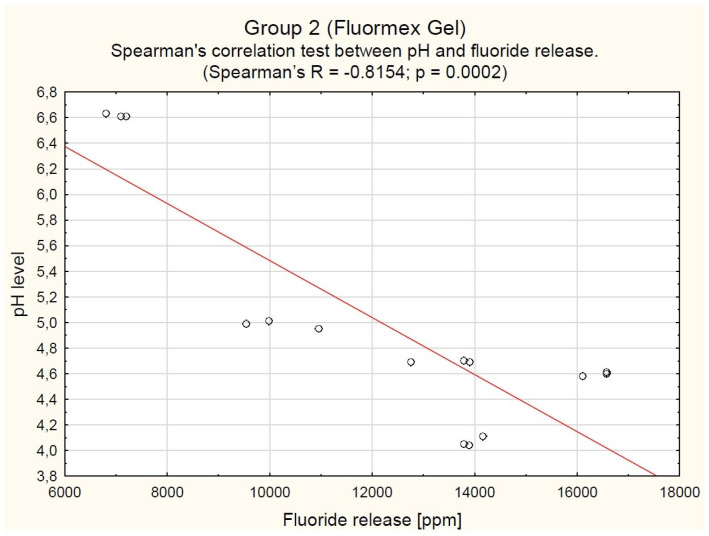
Spearman’s correlation test between pH and fluoride release in Group 2 (Fluormex Gel).

In the G2 (Fluormex) group, a clear inverse relationship was observed, where lower pHs corresponded to greater fluoride release. At the highest pH (6.62 ± 0.01), the fluoride release was 7035 [6899–7100] ppm, while at the lowest pH (4.07 ± 0.04), it peaked at 13,896 [13,896–13,908] ppm (Spearman’s R = −0.8154; *p* = 0.0002; see Figure 3).

In the G3 group (Fluor Protector Gel), a proportional relationship between pHs and fluoride release was noted; however, the relationship was weak and not statistically significant. Fluoride release increased from 5790 [5790–6008] ppm at the lowest pH (5.54 ± 0.01) to 8824.5 [8764–9177] ppm at the highest pH (7.84 ± 0.01) (Spearman’s R = 0.2459; *p* = 0.38).

### 2.4. Discussion

The significance of fluoride in caries protection is indisputable. It has an omnidirectional mechanism of action. Fluoride promotes the incorporation of calcium and phosphate ions into the dental tissue, enhancing remineralization. It also decreases demineralization through the incorporation of fluoride ions into the enamel’s hydroxyapatite via the formation of more acid-stable fluorapatite [29]. Moreover, fluoride ions contribute to the inhibition of bacterial enzymes [30].

Therapeutic levels of fluoride can be achieved from topically applied fluoride products. According to Polish pedodontic dentistry experts, daily application of fluoride gel is an effective form of erosion prevention. They claim that the combination of fluoride toothpaste with fluoride gel increases the chance of reducing tooth decay by 14% compared to using fluoride toothpaste alone [31]. Concentrations of fluoride ions above 0.03 ppm in the tooth environment are sufficient to enhance remineralization [30,31]. That level can be effectively achieved and maintained in the oral cavity via the application of both the Fluormex gel and Fluor Protector Gel analyzed in the presented study. Concentrations of fluoride in artificial saliva with these agents are 5–6 orders of magnitude greater than 0.03 ppm.

As stated in the information from the manufacturer of the dental gels, the concentration of fluoride in 1 g of gel is 8.62 times higher in the Fluormex gel (12,500 ppm) than in Fluor Protector Gel (1450 ppm); see Table 4. Furthermore, they differ in fluoride-containing compounds—amine fluoride and sodium fluoride for Fluormex and potassium fluoride for Fluor Protector Gel. The release of fluoride is greater in all solutions with Fluormex (as suggested by the initial fluoride concentration) with the exception of tap water, where the concentration was higher with Fluor Protector Gel (8824.5 [8764–9177] ppm vs. 7035.0 [6899–7100] ppm). Reference solutions did not contain significant amount of fluoride; hence, they can be considered a reference for all samples without any interactions regarding fluoride concentration. The incubation of both gels in all of the tested solutions resulted in statistically significant elevations in the concentrations of fluoride (*p* < 0.001).

The study by Ravi Kiran et al. confirms the effectiveness of amine fluoride in the prevention of demineralization and white spot lesions [32]. Moreover, this fluoride compound contributes to prevention of plaque accumulation [33]. Likewise, sodium fluoride is an anticaries agent with proven beneficial effects [34]. In their study, Al-Qahtan et al. found that sodium fluoride and potassium fluoride in higher concentrations show a higher acid resistance [35]. This finding may be beneficial in terms of establishing fluoride concentrations for the purposes of caries prevention. The role of sodium and potassium fluoride in caries prevention was also proven in a study by Walther et al., who showed using in vitro tests their potential in limiting the adverse effects of demineralization [36]. This finding may be supported by a study by Alhothali et al., who showed that a single application of 4.1% potassium fluoride significantly inhibited dentin demineralization [37]. The beneficial role of fluoride agents which are contained in both the Fluormex gel and Fluor Protector Gel is proven by numerous studies. In some of them, the concentration of fluoride compounds was considered, and they concluded that higher concentrations are more effective in improving demineralization.

The average pH inside the oral cavity is approximately 6.8 (unstimulated saliva). The action of cariogenic bacteria contributes to the acidification of the oral environment. However, saliva’s pH increases up to 7.8 upon stimulation. This results in greater abilities of buffering acids and facilitates the remineralization of enamel [38]. The role of salivary buffer systems is to maintain the salivary pH at a relatively constant level and decrease the tooth demineralization rate [39]. Saliva plays a vital role in the integrity of the teeth, thanks to its buffering ability and other mechanisms keeping the relationship between the host and oral microbiota in a symbiotic state. A salivary pH below 6.6 is indicative of an increased risk for dental caries [40]. A value equal to or below 6.6 was observed for NaCl and artificial saliva (reference solutions). After incubation with the Fluormex gel, only tap water had a pH of 6.62, which is above 6.6, while for Fluor Protector Gel, incubation in tap water (7.78), demineralized water (6.69), and NaCl (6.62) resulted in values above this limit. The pH level of the oral cavity’s environment is important in terms of its remineralization abilities. The Fluormex gel lowered the pH of all solutions after 48 h of incubation, acidifying the environment to a pH as low as 4.01 in artificial saliva. On the other hand, Fluor Protector Gel did not affect the pH values significantly, with the exception of the incubation in distilled water, where it dropped from 7.15 in the reference solution to 6.31 after incubation (in comparison to the reference solution’s pH). Fluor Protector Gel resulted in a lowering of the pH of both distilled and demineralized water. The possible explanation of this effect could be the low ionic strengths of both liquids. This results in a prioritization of chemical interactions between the ingredients of the fluoride gel matrix; for example, calcium-containing salts can interact with fluoride and result in the precipitation of CaF_2_, which is poorly soluble in water and is linked with inhibiting caries [41,42]. Other interactions, potentially lowering the pH, are possible. However, due to the lack of information on the exact amounts of additives, the exact effect cannot be described.

The critical pH for the enamel’s hydroxyapatite is 5.5, and for fluorapatite, it is 4.5. When the pH decreases below this level, demineralization (dissolution) of the enamel-forming tissues occurs [43]. The pH of the artificial saliva with the Fluormex gel decreased below the level of 4.5 (4.01), which makes the tissue vulnerable to demineralization. At the same time, the pH of the artificial saliva with Fluor Protector Gel presents a value higher than 5.5 (5.58), which is above the critical point both for hydroxyapatite and fluorapatite.

Tooth decay is a very common multifactorial disease, affecting both the deciduous and permanent dentition of patients at different ages, caused by acids produced by bacteria. From the clinical point of view, both fluoride ion concentrations and the saliva’s pH are crucial in terms of caries development. With an adequate concentration of calcium, phosphate, and fluoride ions, enamel demineralization can be reversed [44]. In this study, only one sample of each type of the material was used. The behavior of biomaterials inside the oral cavity is a complex issue. In order to transfer the results presented in the study to real-world application, studies on real patients need to be conducted to compare the amounts of fluoride involved in dental prophylaxis and caries development/inhibition.

## 3. Conclusions

This study highlights the vital role of fluoride in caries prevention. It underscores the importance of maintaining fluoride concentrations above 0.03 ppm to optimize enamel health, a level achievable with the application of fluoride gels like Fluormex and Fluor Protector Gel. The results of the above study led to the conclusions that fluoride ion release by fluoride gels is dependent on the incubation liquid, the composition of fluoride gels affects the degree of fluoride ion release, and fluoride gels containing a lower fluoride concentration are capable of releasing proportionally more fluoride ions than gels with a higher concentration.

The findings suggest that while both gels can effectively provide therapeutic levels of fluoride, their impacts on the oral environment differ significantly. The potential for the Fluormex gel to lower the pH to demineralization levels highlights the need for careful consideration of its use. Further clinical studies on real patients are necessary to validate these findings and establish optimized protocols for fluoride application in dental prophylaxis and caries management. This may contribute to enabling the use of a more individualized fluoride prophylaxis.

The limitations of the presented study include the limited number of evaluated commercial gels. Further studies using a larger number of fluoride gels containing the same fluoride-releasing media as well as in vivo studies assessing the time required to return to the proper oral pH after the application of pH-lowering preparations are recommended to confirm the conclusions drawn.

## 4. Materials and Methods

### 4.1. Incubation Liquids

Studies regarding fluoride release and pH assessment were conducted using five different liquids, namely tap water, distilled water, demineralized water, 0.9% NaCl solution in demineralized water, and artificial saliva (based on demineralized water). Origin and composition of aforementioned liquids/solutions is presented in Table 5.

### 4.2. Fluoride Gels

**Figure 4:**
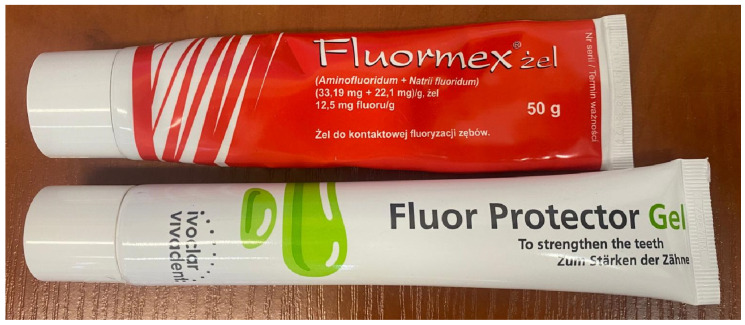
Fluormex and Fluor Protector Gel—commercial fluoride gels used in study.

Studies were conducted on two commercially available fluoride gels: Fluormex (CHEMA-ELEKTROMET, Rzeszów, Poland) and Fluor Protector Gel (Ivoclar Vivadent, Schaan, Liechtenstein). Used products are depicted in Figure 4. Used products are depicted in Figure 4 (Photo taken by M.D. in Department of Pediatric Dentistry and Preclinical Dentistry, Wroclaw Medical University, for academic research only).

The gels utilized in this study exhibited notable differences in their composition, particularly with respect to the type and concentration of fluoride compounds, with Fluormex gel demonstrating a higher fluoride concentration. Fluormex gel contains amine fluoride and sodium fluoride while Fluor Protector is based on potassium fluoride as a source of fluoride ions. Furthermore, the formulations varied in the presence of additional substances, which may influence their clinical properties and efficacy. A comprehensive summary of the gel compositions is provided in Table 4. These two commercial products were included in the study due to their popularity among dental clinicians. They represent fluoride gels containing different fluorine compounds which may be relevant in context of fluoride release. Other studies can be found in the literature which compare different fluoride gels (Miradent Mirafluor—gel and Elmex Gel [23]; Nupro APF gel [45]).

### 4.3. Fluoride Release and pH Assesment

**Figure 5:**
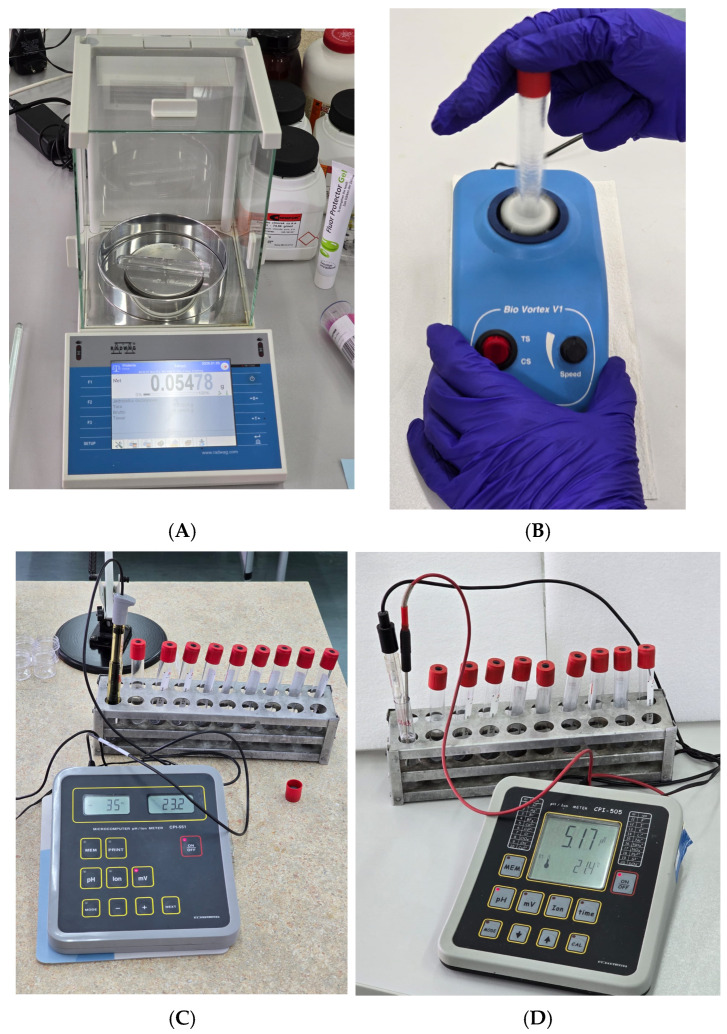
Polypropylene tube with fluoride gel on scale (**A**); fluoride gel sample vortexed with solution (**B**); 9609 Orion selective electrode coupled with CP-551 processing unit for measurement of fluoride concentration (**C**); and ESAgP-303W electrode connected to CPI-505 pH-meter for measuring pH (**D**).

A total of 0.055 g of each commercial fluoride gel was placed in 5 separate polypropylene tubes for each gel (Figure 5A). Subsequently, 5.5 mL of each solution from Table 5 were added to the tubes, vortexed using Bio Vortex V1 (Biosan, Riga, Latvia, Figure 5B), and magnetically stirred (500 RPM). Number of released F^−^ ions was registered using 9609 Orion selective electrode (Thermo Scientific, Waltham, MA, USA) coupled with CP-551 processing unit (Elmetron, Zabrze, Poland) after 48 h of incubation (Figure 5C). The pH of incubation solutions was assessed before incubation and after 48 h using ESAgP-303W electrode (Eurosensor, Gliwice, Poland) connected to CPI-505 pH-meter (Elmetron) in three (N = 3) repetitions for each solution (Figure 5D). Fluoride release was measured in ten measurements for each solution (N = 10). Methodology of comparing commercial fluoride gels in various solutions was adapted from article by Turkalj et al. [23].

### 4.4. Statistical Analysis

The normality of the data was assessed using the Kolmogorov–Smirnov test. Fluoride concentrations for the Reference, Fluormex, and Fluor Protector Gel solutions were analyzed using the Kruskal–Wallis ANOVA, followed by Dunn’s Multiple Comparison Test for post hoc analysis. pH levels were assessed using one-way ANOVA with Tukey’s post hoc test. The correlation between fluoride release and pH levels across groups was checked using Spearman’s rank correlation test. Statistical analyses were performed using Statistica software (version 13.3.721.1, StatSoft, Tulsa, OK, USA). A *p*-value of less than 0.05 was considered statistically significant. Lower and upper quartiles are presented in the square brackets in the text.

## Figures and Tables

**Table 1:** Sources from the literature on fluoride gel behavior, presented along with the aims of the studies.

Aim of the Study	Reference
Determination of fluoride ion concentrations in different solutions from gels with different matrices.	[23]
Evaluation of fluoride ion concentrations in saliva after brushing teeth with gel with and without subsequent mouth rinsing with water.	[27]
Evaluation of the fluoride concentration in saliva after application of Sultan acidified phosphate fluoride (APF) gel, Sultan foam, and Cina gel at different time intervals.	[24]
Comparison of fluoride ion concentrations in saliva after application of fluoride varnish, solution, and gel at different time intervals.	[25]
Comparison of fluoride release from varnish, SDF, APF gel, and artificial saliva.	[26]
Evaluation of the effect of different preparations of fluoride gels on the salivary pH of albino rats	[28]

**Table 2:** Fluoride release—intra-group comparison (ANOVA Kruskal–Wallis; H (4, n = 50), *p* = 0.0000).

Groups	Solutions		N	Median [ppm]	Lower Quartiles [ppm]	Top Quartiles [ppm]	Interquartile Range [ppm]	*p* Value
G1 Reference	1	Tap water	10	0.22	0.20	0.23	0.03	1 vs. 3: *p* = 0.0000 1 vs. 4: *p* = 0.0000 1 vs. 5: *p* = 0.0000 2 vs. 3: *p* = 0.0216 2 vs. 4: *p* = 0.0216 2 vs. 5: *p* = 0.0216
	2	Distilled water	10	0.10	0.10	0.11	0.01	
	3	Demineralized water	10	0	0	0	0	
	4	NaCl	10	0	0	0	0	
	5	Artificial Saliva	10	0	0	0	0	
G2 Fluormex	1	Tap water	10	7035	6899	7100	201	1 vs. 2: *p* = 0.0059 1 vs. 3: *p* = 0.0000 3 vs. 4: *p* = 0.0000
	2	Distilled water	10	13,666	12,570	13,900	1330	
	3	Demineralized water	10	16,917	16,582	17,090	508	
	4	NaCl	10	9893	9541	9893	352	
	5	Artificial Saliva	10	13,896	13,896	13,908	12	
G3 Fluor Protector Gel	1	Tap water	10	8824.5	8764	9177	413	1 vs. 4: *p* = 0.0007 1 vs. 5: *p* = 0.0205 2 vs. 4: *p* = 0.0000 2 vs. 5: *p* = 0.0003 3 vs. 4: *p* = 0.0015 3 vs. 5: *p* = 0.0393
	2	Distilled water	10	9188	9177	9877	700	
	3	Demineralized water	10	8999	8564	9141	577	
	4	NaCl	10	5755	5529	5790	261	
	5	Artificial saliva	10	5790	5790	6008	218	

**Table 3:** pHs of reference incubation solutions as well as those of Fluormex and Fluor Protector Gel commercial fluoride gels after 48 h of incubation (one-way ANOVA with Tukey’s post hoc test).

Solution		N	pH G1 Reference Mean ± SD	pH G2 Fluormex Mean ± SD	pH G3 Fluor Protector Mean ± SD	*p* Value
1	Tap water	3	7.61 ± 0.03	6.62 ± 0.01	7.84 ± 0.01	G1 vs. G2 vs. G3 *p* = 0.0002
2	Distilled water	3	7.15 ± 0.07	4.69 ± 0.01	6.31 ± 0.06	G1 vs. G2 vs. G3 *p* = 0.0002
3	Demineralized water	3	6.73 ± 0.01	4.60 ± 0.02	6.65 ± 0.04	G1 vs. G2. G2 vs. G3 *p* = 0.0002 G1 vs. G3 *p* = 0.0329
4	NaCl	3	6.49 ± 0.04	4.99 ± 0.03	6.62 ± 0.01	G1 vs. G2. G2 vs. G3 *p* = 0.0002 G1 vs. G3 *p* = 0.0054
5	Artificial Saliva	3	5.04 ± 0.02	4.07 ± 0.04	5.54 ± 0.01	G1 vs. G2 vs. G3 *p* = 0.0002
*p* value			1 vs. 2 vs. 3 vs. 4 vs. 5 *p* = 0.0002	1 vs. 2 vs. 3 vs. 4 vs. 5 *p* = 0.0002	1 vs. 2 vs. 3 vs. 5 3,4 vs. 5 *p* = 0.0002 3 vs. 4 *p* = 0.8163	-

**Table 4:** Detailed characteristics of gels.

Fluoride Gel	Lot Number	Fluorine Compound	Fluoride Concentration in 1 g of a Gel	Additives
Fluormex—gel (CHEMA-ELEKTROMET, Rzeszów, Poland)	230302	amine fluoride and sodium fluoride	12.5 mg (12,500 ppm)	glycerol, propyl parahydroxybenzoate, methyl parahydroxybenzoate, hydroxyethylcellulose, polyoxyethylene polyoxypropylene glycol, purified water, mint flavoring
Fluor Protector—gel (Ivoclar Vivadent, Schaan, Liechtenstein)	Z06YHM	potassium fluoride	1.45 mg (1450 ppm)	xylitol, calcium glycerophosphate, D-panthenol, flavoring agents, stabilizers

**Table 5:** Incubation liquids utilized in study; pH presented as a mean value.

No.	Solution	pH	Origin/Supplier/Composition
1	Tap water	7.61	Sourced from ul. Krakowska 26, Wrocław, Poland
2	Distilled water	7.15	Sourced from commercial water distiller
3	Demineralized water	6.73	Supplied by Euromex (Krotoszowice, Poland)
4	NaCl *	6.49	0.9% NaCl solution based on demineralized water from pt.3
5	Artificial saliva *	5.04	Solution based on demineralized water from pt.3 containing 28.63% of urea, 11.45% of NaCl, 11.45% of KCl, 26.00% of CaCl_2_·2H_2_O, 22.33% of NaH_2_PO_4_·2H_2_O and 0.14% of Na_2_S·9H_2_O

* NaCl, KCl, Na_2_S·9H_2_O, NaH_2_PO_4_·2H_2_O and urea were manufactured by Chempur (Piekary Śląskie, Poland); CaCl_2_·2H_2_O was supplied by Sigma Aldrich (St. Louis, MO, USA).

## Data Availability

Data are contained within the article.
